# Vaccination status and outcomes of COVID‐19 patients admitted to a tertiary hospital in Iran during the dominant Delta variant period

**DOI:** 10.1002/iid3.790

**Published:** 2023-02-24

**Authors:** Hamed Mehdinezhad, Bardia Karim, Niloufar Ahmadi, Reza Mohseni Ahangar, Ali Asadolahzadeh, Mahmoud Sadeghi Haddad‐Zavareh, Fatemeh khoshkhou, Zeynab Qolami, Neda Mahdinezhad Gorji, Mouloud Agajani Delavar

**Affiliations:** ^1^ Department of Internal Medicine, School of Medicine, Rouhani Hospital Babol University of Medical Sciences Babol Mazandaran Iran; ^2^ Department of Internal Medicine Babol University of Medical Sciences Babol Mazandaran Iran; ^3^ Department of Infectious Disease, School of Medicine, Infectious Diseases and Tropical Medicine Research Center, Health Research Institute Rouhani Hospital Babol University of Medical Sciences Babol Mazandaran Iran; ^4^ Department of Internal Medicine, Infertility and Reproductive Health Research Center, Health Research Institute Babol University of Medical Sciences Babol Mazandaran Iran

**Keywords:** breakthrough infection, COVID‐19 vaccines, hospitalization, Iran, mortality, pandemic

## Abstract

**Background:**

This study aimed to determine the characteristics, vaccination status, and outcomes of confidence interval (COVID‐19) patients, admitted to a tertiary hospital in Iran during the predominant severe acute respiratory syndrome‐coronavirus 2 (SARS‐CoV‐2) Delta variant period.

**Methods:**

This retrospective study assessed the medical records of all hospitalized COVID‐19 patients, who were admitted to a tertiary hospital from July 10 to September 15, 2021. Adjusted binary logistic regression analyses were conducted to determine factors associated with poor outcomes.

**Results:**

More than 25% of hospitalized patients received at least one vaccine dose of SARS‐CoV‐2. The Sinopharm BIBP vaccine (China) was the most commonly received vaccine (73.3%). After adjusting for age and comorbidities, the adjusted odds ratio (AOR) for poor outcomes was significantly lower in hospitalized patients who received Remdesivir compared to those not receiving Remdesivir (AOR: 0.35; 95% confidence interval [CI]: 0.15, 0.78; *p* < .010). Besides, age ≥50 years (AOR: 2.51; 95% CI: 1.38, 4.59; *p* < .003), low educational level (AOR: 3.99; 95% CI: 1.17, 13.53; *p* < .027), work outside in the past year (AOR: 1.75; 95% CI: 1.02, 3.00; *p* < .041), and diabetes mellitus (AOR: 1.95; 95% CI: 1.66, 3.26; *p* = .011) were associated with more poor outcomes.

**Conclusion:**

Based on the present results, the risk of mortality and the risk of poor outcomes were lower in patients who received Remdesivir compared to those not receiving Remdesivir. The number of vaccinated patients was smaller than the unvaccinated among hospitalized patients. It is important to emphasize that vaccination reduced the need for hospitalization and that only vaccinated patients with comorbidities required hospitalization.

## Graphical abstract

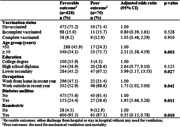

## 1 INTRODUCTION

The coronavirus disease 2019 (COVID‐19), caused by severe acute respiratory syndrome‐coronavirus 2 (SARS‐CoV‐2), was first reported in Wuhan, China in December 2019^1^, ^2^ and spread rapidly around the world.^3^ In Iran, the first case of COVID‐19 was registered on February 18, 2020^4^; since then, Iran has faced several surges of this disease. The fifth wave of COVID‐19, caused by the Delta variant of SARS‐CoV‐2, started on July 10, 2021. Several vaccines have been introduced and approved in clinical trials since December 2020.^5^, ^6^, ^7^, ^8^ The vaccination program against COVID‐19 first started with the Pfizer‐BioNTech vaccine on December 16, 2020 and with the Moderna vaccine on December 28, 2020 for healthcare workers in the United States.^9^ In Iran, vaccination against COVID‐19 was initiated with the Russian Sputnik V vaccine on February 23, 2021 and continued with the Sputnik V, Oxford‐AstraZeneca (imported from Japan, Italy, and Korea), Sinopharm BIBP (China), PastoCovac (in the clinical trial phase), Barekat (in the clinical trial phase), and Iran‐Cuba joint vaccines (Pasteur Institute in collaboration with Cuba). According to the Iranian Ministry of Health and Medical Education, before the fifth wave of COVID‐19 in Iran, nearly 4,436,083 people had received the first dose of COVID‐19 vaccine, and 1,596,507 people had received the second dose; the total number of received vaccines was estimated at 6,132,590 doses in Iran.^10^, ^11^

Studies suggest that COVID‐19 vaccination can reduce the risk of mortality and hospitalization due to this disease.^12^, ^13^ Therefore, mass vaccination, in combination with the existing control measures, is one of the main elements of epidemic control. Although the findings of clinical trials are encouraging, accurate information on vaccines is scarce, especially for determining vaccine efficacy in specific ethnic groups.^14^, ^15^ In this regard, a study conducted on 187 COVID‐19 patients, aged ≥65 years, admitted to an Israeli hospital, reported an adjusted association between the vaccination status and hospitalization in the elderly.^16^ In addition, several investigators have shown a positive relationship between older age and higher viral load and it might be as indicative for disease severity of COVID‐19 in elderly.^17^, ^18^ According to these findings, the present study aimed to determine the characteristics and outcomes of vaccinated and nonvaccinated COVID‐19 patients and to measure the adjusted odds ratios for poor outcomes in hospitalized patients, admitted to a tertiary hospital in Iran during the dominant Delta variant period.

## 2 MATERIALS AND METHODS

This retrospective study assessed the vaccination status and outcomes of hospitalized COVID‐19 patients, admitted to a tertiary hospital in Iran, with a 300‐bed capacity (and 25 intensive care unit beds) during the dominant Delta variant period. From July 10 to September 15, 2021, a total of 4650 patients with a suspicion of COVID‐19 were examined in this center. Nearly 700 patients with a confirmed diagnosis of active COVID‐19, according to both real‐time polymerase chain reaction (RT‐PCR) and chest computed tomography (CT) scan, were admitted to this hospital.

The medical records of all hospitalized COVID‐19 patients were assessed in this study, and 698 hospitalized cases were selected. Initially, data, including the demographic characteristics (e.g., age, sex, residence, educational level, occupation, and marital status), comorbidities (e.g., cardiovascular disease, hypertension, hyperlipidemia, and diabetes), oxygen saturation upon hospital admission, vaccination status (e.g., date of first dose of vaccine, date of second dose, and type of vaccine), mechanical ventilation, and outcomes of patients (e.g., type of treatment, type of medication received with or without mechanical ventilation, and length of hospitalization) were recorded from the patients' medical records in a predefined google spreadsheet checklist. If information on some items was incomplete, the checklist was completed through interview with the patient or the patient's family.

Information regarding the vaccination of patients was collected from the Iranian Immunization Electronic Registry System. The vaccination status was divided into four categories: (1) unvaccinated, not receiving a COVID‐19 vaccine; (2) incompletely vaccinated, receiving one dose of vaccine ≥2 weeks before the disease onset; (3) receiving the second dose <2 weeks before the disease onset; and (4) fully vaccinated, receiving the second dose ≥2 weeks before the disease onset (10). The outcomes of patients were divided into two categories. Poor outcomes were defined as the need for mechanical ventilation and mortality, and favorable outcomes were defined as either discharge from hospital or stay in hospital without any need for ventilation.

SPSS version 20 was used for data analysis. For statistical analysis, categorical variables were compared between patients with and without vaccination, using Chi‐square and Fisher's exact test, and quantitative variables were compared using one‐way analysis of variance. Moreover, adjusted binary logistic regression analyses were conducted for factors associated with poor outcomes.

## 3 RESULTS

Between July 15 and September 15, 2021, a total of 698 patients with COVID‐19, including 415 (59.5%) women and 283 (40.5%) men, were admitted to our hospital during the predominant Delta variant period. The mean age of women was 52.7 ± 16.1 years, and the mean age of men was 53.4 ± 16.7 years. Regarding the vaccination status of hospitalized COVID‐19 patients, 522 (74.8%) had not received any vaccines, while 176 (25.2%) patients had received at least one dose of SARS‐CoV‐2 vaccine. Among these patients, 129 (73.3%) had been vaccinated with the Sinopharm vaccine, 22 (12.5%) with the AstraZeneca vaccine, and 15 (8.5%) with Barekat vaccine (Table 1).

**Table 1:** The vaccination status of COVID‐19 patients admitted to a tertiary hospital in Iran during the dominant delta variant period.

	No. of cases	Percent
Vaccination status (*n* = 698)		
Fully vaccinated^a^	67	9.6
Partially vaccinated^b^	81	11.6
Single‐dose vaccinated <2 weeks before illness onset	28	4.0
Unvaccinated	522	74.8
Vaccine type, if vaccinated (*n* = 176)		
Sinopharm BIBP vaccine (made in China)	129	73.3
Oxford‐AstraZeneca (imported from Japan, Italy, and Korea)	22	12.5
Barekat (in phase of clinical trials)	15	8.5
Pastocovac (in phase of clinical trials)	7	4.0
Sputnik V vaccine (made in China)	3	1.7

^a^ Fully vaccinated: the second dose vaccinated ≥2 weeks before illness onset.

^b^ Partially vaccinated: receipt 1 dose of vaccine ≥ 2 weeks before illness onset or receipt of second dose <2 weeks before illness onset.

Considering the demographic characteristics of the patients, a significant difference was found between age and education level with vaccination status (*p* < .001). There was no significant difference between other demographic characteristics of the patients and vaccination status (Table 2).

**Table 2:** Comparable demographic between unvaccinated and vaccinated of COVID‐19 patients admitted to a tertiary hospital in Iran during the dominant delta variant period.

	Unvaccinated (*n* = 522) *n* (%)	Incomplete vaccinated^a^ (*n* = 109) *n* (%)	Complete vaccinated^b^ (*n* = 67) *n* (%)	*p*‐value
Age (years), mean (*SD*)	49.8 (15.3)	59.0 (13.8)	68.1 (16.4)	<.001
Age group (years)				<.001
<50	273 (52.3)	22 (20.2)	10 (14.9)	
≥50	249 (47.7)	87 (79.8)	57 (85.1)	
Sex				.398
Male	204 (39.1)	49 (45.0)	30 (44.8)	
Female	318 (60.9)	60 (55.0)	37 (55.2)	
Residence				.877
Urban	302 (57.9)	61 (56.0)	37 (55.2)	
Rural	220 (42.1)	48 (44.0)	30 (44.8)	
Education				<.001
Lower secondary	89 (17.0)	8 (7.3)	6 (9.0)	
High school diploma	217 (41.6)	28 (25.7)	19 (28.4)	
College degree	216 (41.4)	73 (67.0)	42 (62.6)	
Occupation				.149
Work from home in recent year	245 (46.9)	50 (45.9)	23 (34.3)	
Work outside in recent year	277 (53.1)	59 (54.1)	44 (65.7)	
Marital status				.205
Married	474 (90.8)	101 (92.7)	65 (97.0)	
Single	48 (9.2)	8 (7.3)	2 (3.0)	

^a^ Incomplete vaccinated: single‐dose vaccinated <2 weeks before COVID‐like illness onset or single‐dose vaccinated ≥2 weeks before COVID‐like illness onset or second dose vaccinated <2 weeks before COVID‐like illness onset.

^b^ Complete vaccinated: the second dose vaccinated ≥2 weeks before COVID‐like illness onset.

The total number of hospitalized patients who expired during the study was 34 (4.9%). The mean age of deceased patients was significantly higher than that of recovered patients (58.9 ± 17.0 vs. 52.2 ± 15.9 years). Overall, the rate of poor outcomes was 10.0% (*n* = 70) in hospitalized patients. The mean oxygen saturation upon hospital admission was 91.0 ± 6.4%. The majority of the patients (92.1%) received oxygen by mask or high‐flow nasal cannula. The number of patients with both noninvasive and invasive mechanical ventilation was 55 (7.9%). Moreover, the mean length of hospital stay was 6.5 ± 4.3 days among the patients. There was no significant association between the vaccination status of patients and oxygen saturation upon hospital admission, mechanical ventilation, length of hospital stay, poor outcomes, and mortality.

Based on the findings, at least one comorbid disease was reported in 54.7% (*n* = 382) of the patients. The number of patients with full vaccination and at least one comorbidity was significantly higher than that of patients with incomplete or no vaccination (*p* < .001). The most common comorbidities were hypertension (28.2%), diabetes mellitus (25.8%), and hyperlipidemia (12.8%). The results showed that the number of fully vaccinated patients with diabetes mellitus (*p* < .001), hypertension (*p* < .001), and cardiovascular disease (*p* = .004) was significantly higher than that of patients with incomplete or no vaccination. However, there was no significant association between the vaccination status and other comorbidities (Table 3).

**Table 3:** Comparable comorbidities and outcome of COVID‐19 patients admitted to a tertiary hospital in Iran during the dominant Delta variant period.

	Unvaccinated (*n* = 522) *n* (%)	Incomplete vaccinated^a^ (*n* = 109) *n* (%)	Complete vaccinated^b^ (*n* = 67) *n* (%)	*p*‐value
Hospitalization days, mean (*SD*)	6.3 (3.8)	7.1 (6.3)	6.4 (3.9)	.241
Hospitalization days				.988
≤5	286 (54.8)	59 (54.1)	37 (55.2)	
>5	236 (45.2)	50 (45.9)	30 (44.8)	
Comorbidities				<.001
No	265 (50.8)	37 (33.9)	14 (20.9)	
Yes	257 (49.2)	72 (66.1)	53 (79.1)	
Cardiovascular disease				.004
No	448 (85.8)	89 (81.7)	47 (70.1)	
Yes	74 (14.2)	20 (18.3)	20 (29.9)	
Hypertension				<.001
No	403 (77.2)	68 (62.4)	30 (44.8)	
Yes	119 (22.8)	41 (37.6)	37 (55.2)	
Hyperlipidemia				.152
No	462 (88.5)	93 (85.3)	54 (80.6)	
Yes	60 (11.5)	16 (14.7)	13 (19.4)	
Diabetes mellitus				<.001
No	412 (78.9)	66 (60.6)	40 (59.7)	
Yes	110 (21.1)	43 (39.4)	27 (40.3)	
Oxygen saturation at hospital admission (%)				.158
<90	354 (67.8)	69 (63.3)	38 (56.7)	
≥90	168 (32.2)	40 (36.7)	29 (43.3)	
Mechanical ventilation				.927
No	481 (92.1)	101 (92.7)	61 (91.0)	
Yes	41 (7.9)	8 (7.3)	6 (9.0)	
Outcome				.613
Favorable outcome	472 (90.4)	98 (89.9)	58 (86.6)	
Poor outcome	50 (9.6)	11 (10.1)	9 (3.4)	
Consequence of the patient				.242
Recovered	498 (95.4)	105 (96.3)	61 (91.0)	
Died	24 (4.6)	4 (3.7)	6 (9.0)	

^a^ Incomplete vaccinated: single‐dose vaccinated <2 weeks before COVID‐like illness onset or single‐dose vaccinated ≥2 weeks before COVID‐like illness onset or second dose vaccinated <2 weeks before COVID‐like illness onset.

^b^ Complete vaccinated: the second dose vaccinated ≥2 weeks before COVID‐like illness onset.

The medications used to treat COVID‐19 in hospitalized patients included Remdesivir for 661 (94.7%) patients, Favipiravir for 9 (1.3%) patients, glucocorticoids with dexamethasone for 653 (93.6%) patients, and an anticoagulant with an unfractionated heparin/low‐molecular‐weight heparin (UFH/LMWH) for 664 (95.1%) patients. After adjusting for age, education level, and comorbidities, the adjusted odds ratio (AOR) for poor outcomes was significantly lower in hospitalized patients receiving Remdesivir compared to patients not receiving Remdesivir (AOR: 0.35; 95% confidence interval [CI]: 0.15, 0.78; *p* < .010). Additionally, age group ≥50 years (AOR: 2.51; 95% CI: 1.38, 4.59; *p* < .003), low education (AOR: 3.99; 95% CI: 1.17, 13.53; *p* < .027), work outside in the last year (AOR: 1.75; 95% CI: 1.02, 3.00; *p* < .041), and diabetes mellitus (AOR: 1.95; 95% CI: 1.66, 3.26; *p* = .011) were associated with poorer outcomes in hospitalized patients. However, no significant association was found between poor outcomes and vaccination status, sex, comorbidities, cardiovascular disease, hypertension, and hyperlipidemia (Table 4).

**Table 4:** Adjusted odds risk for poor outcome of COVID‐19 patients admitted to a tertiary hospital in Iran during the dominant Delta variant period.

	Favorable outcome^a^ (*n* = 628) *n* (%)	Poor outcome^b^ (*n* = 70) *n* (%)	Adjusted odds ratio (95% CI)	*p*‐value
Vaccination status				
Unvaccinated	472 (75.2)	50 (71.4)	1.00	
Incomplete vaccinated	98 (15.0)	11 (15.7)	0.80 (0.39, 1.61)	.528
Complete vaccinated	58 (9.2)	9 (12.9)	1.05 (0.48, 2.29)	.910
Age group (years)				
<50	288 (45.9)	17 (24.3)	1.00	
≥50	340 (54.1)	53 (75.7)	2.51 (1.38, 4.59)	.003
Sex				
Male	260 (41.4)	23 (32.9)	1.00	
Female	368 (58.6)	47 (67.1)	1.44 (0.86, 2.44)	.169
Residence				
Urban	362 (57.6)	38 (54.3)	1.00	
Rural	266 (42.4)	32 (54.7)	1.11 (0.67, 1.48)	.677
Education				
College degree	100 (15.9)	3 (4.3)	1.00	
High school diploma	244 (38.9)	20 (28.6)	2.64 (0.77, 9.10)	.125
Lower secondary	284 (45.2)	47 (67.1)	3.99 (1.17, 13.53)	.027
Occupation				
Work from home in recent year	296 (47.1)	22 (31.4)	1.00	
Work outside in recent year	332 (52.9)	48 (68.6)	1.75 (1.02, 3.00)	.041
Marital status				
Married	576 (91.7)	64 (91.4)	1.00	.465
Single	52 (8.3)	6 (8.6)	0.712 (0.29, 1.77)	
Comorbidities				
No	291 (46.3)	25 (35.7)	1.00	
Yes	337 (53.7)	45 (64.3)	1.14 (0.66, 1.97)	.636
Cardiovascular disease				
No	529 (84.2)	55 (78.6)	1.00	
Yes	99 (15.8)	15 (21.4)	1.08 (0.58, 2.03)	.811
Hypertension				
No	548 (72.9)	43 (61.4)	1.00	
Yes	170 (27.1)	27 (38.6)	1.196 (0.69, 2.07)	.523
Hyperlipidemia				
No	548 (87.3)	61 (87.1)	1.00	
Yes	80 (12.7)	9 (12.9)	0.81 (0.39, 1.72)	.588
Diabetes mellitus				
No	475 (75.6)	43 (61.4)	1.00	
Yes	153 (24.4)	27 (38.6)	1.95 (1.66, 3.26)	.011
Remdesivir				
No	28 (4.5)	9 (12.9)	1.00	
Yes	600 (95.5)	61 (87.1)	0.35 (0.15, 0.78)	.010

^a^ Favorable outcome: either discharge from the hospital or stay in the hospital without any need for ventilation.

^b^ Poor outcomes: the need for mechanical ventilation and mortality.

## 4 DISCUSSION

In this study, only 9.6% of hospitalized COVID‐19 patients were fully vaccinated. Vaccination against COVID‐19 first started on February 10, 2020 in Iran.^19^ Delayed vaccination was due to several fundamental challenges related to the medical staff, as well as political sanctions and poor economic status of the county.^10^ The demographic characteristics, including sex, place of residence, occupation, and marital status of all severe and moderate COVID‐19 cases with positive RT‐PCR results, who were admitted to our tertiary hospital in Iran from July to September 2021, were similar. It was observed that older age, high level education (college degree), comorbidities, hypertension, and diabetes were higher in the fully vaccinated group compared to the unvaccinated group, which might be related to the COVID‐19 vaccination program. It is worth mentioning that vaccination in Iran was initiated by prioritizing the medical staff and then high‐risk groups, including patients with underlying diseases and the elderly.^20^

Moreover, the present findings showed that the risk of poor outcomes was similar in COVID‐19 patients who had received both vaccine doses and those who had no received any vaccines or only a single‐dose of Sinopharm BIBP, Oxford‐AstraZeneca, Barekat, PastoCovac, or Sputnik V vaccine. Besides, our findings did not indicate any significant difference in terms of poor outcomes between younger and older hospitalized patients. The higher rate of poor outcomes in patients with comorbidities suggests that the presence of comorbidities is associated with an increased risk and frequent exacerbation of infection.^21^, ^22^, ^23^ Additionally, the mortality rate was 4.9% in our hospitalized patients. No significant difference was found in the mortality rate between fully vaccinated and unvaccinated patients in this study. This finding is consistent with the results of several cohort studies, which reported that the mortality rates were similar in vaccinated and unvaccinated hospitalized patients with COVID‐19.^14^, ^24^, ^25^

On the other hand, several studies have reported that COVID‐19 vaccination is highly effective in reducing the rate of hospitalization and improving the outcomes of COVID‐19 patients.^16^, ^26^, ^27^ Consistent with these studies, Özüdoğru et al.^27^ showed that in Turkey, when the Delta variant was dominant, 100% of people with full vaccination and 94% of people with incomplete vaccination did not require hospitalization. Also, death and need for mechanical ventilation were not observed in fully vaccinated hospitalized patients. Moreover, the mean length of hospital stay was shorter in patients with incomplete vaccination compared to those who were unvaccinated (10 vs. 7 days).^27^

However, the current study did not indicate a significant difference in the length of hospital stay between fully vaccinated and unvaccinated patients (average of 6 days in both groups). A possible explanation for this discrepancy is the type of COVID‐19 vaccine. People in Turkey received BioNTech or CoronaVac vaccine. Tenforde et al. showed that the rate of vaccination with an mRNA COVID‐19 vaccine (Pfizer/BioNTech) was significantly lower in hospitalized COVID‐19 patients with invasive mechanical ventilation or progression to death.^28^ In the current study, 73.3% of hospitalized patients had received an inactivated vaccine (Sinopharm). In addition the fully vaccinated group had a higher percentage of patients with comorbidities. Another explanation for this discrepancy is that some vaccinated people might have mistakenly assumed that they were fully vaccinated against COVID‐19 and therefore, reduced their adherence to safety and health protocols.^29^

According to the present results, the rates of mortality and poor outcomes in hospitalized patients were lower in the fifth wave of the pandemic compared to the other waves of COVID‐19. A possible explanation for the relative decline in the mortality rate and poor outcomes during this period may involve several factors, including updated evidence‐based COVID‐19 protocols according to the World Health Organization guidelines and experiences of our hospital team.^30^, ^31^ During the fifth wave of COVID‐19, in our hospital, the treatment of choice included medications, such as Remdesivir, glucocorticoids, and UFH/LMWH for the treatment of almost all hospitalized patients. Around 95% of our hospitalized patients received Remdesivir. The adjusted risks of mortality and poor outcomes were 83% and 65% lower in hospitalized patients who received Remdesivir compared to those not receiving Remdesivir, respectively. Several studies suggest that Remdesivir is effective in the treatment of hospitalized patients with COVID‐19.^32^, ^33^, ^34^

There are several limitations to this study. First, this study examined the vaccination status and outcomes of COVID‐19 patients during the dominant Delta variant period with a relatively small number of patients. Second, this study used the data of COVID‐19 patients, referred to Ayatollah Rohani Hospital of Babol, affiliated to Babol University of Medical Sciences, Babol, Iran. Although this hospital is the largest treatment center in Babol, future studies based on the data of the general population can provide stronger evidence on the protective effects of COVID‐19 vaccination. Third, the majority of patients, especially the elderly, received Sinopharm BIBP vaccine (China), while a small number of patients received four other types of vaccine. This study could not analyze the type of vaccine, which might have influenced the outcomes of COVID‐19 patients, admitted to the hospital during the Delta variant‐dominant period.

Conversely, the strengths of this study were the lack of diagnosis bias due to RT‐PCR assays and lung CT scans for diagnosing COVID‐19 in almost all hospitalized cases. Also, all hospitalized patients during the dominant Delta variant period admitted to our hospital were included in this study; therefore, we can argue that the study population was a representative sample. Besides, in this study, some potential confounders were controlled. Finally, information on the vaccination of all hospitalized patients was collected from the Iranian Immunization Electronic Registry System; therefore, there was no selection bias in categorization of vaccination.

## 5 CONCLUSION

The findings of the present study indicated that age >50 years and low education were associated with significantly poorer outcomes. The risk of mortality (83%) and the risk of poor outcomes (65%) were lower in patients who received Remdesivir compared to those not receiving Remdesivir. In addition, we observed the rates of hospitalization and mortality was similar in vaccinated and unvaccinated patients. Nevertheless, the efficacy of COVID‐19 vaccination was not confirmed, as only 9.6% of the total hospitalized COVID‐19 patients (*n* = 698) were fully vaccinated. Besides, the fully vaccinated group included a higher percentage of patients with comorbidities. One explanation for the smaller number of vaccinated patients admitted to the hospital could be the reduced need for hospitalization due to vaccination, as only vaccinated individuals with comorbidities required hospitalization. Finally, the percentage of vaccinated patients during the study, as well as the type of vaccine, varied in different age groups. Almost two‐thirds of patients had received Sinopharm BIBP vaccine (China), which might be attributed to the inefficacy of this vaccine.

## AUTHOR CONTRIBUTIONS

**Hamed Mehdinezhad**: conceptualization; formal analysis; investigation; methodology; project administration; validation; writing – original draft. **Bardia Karim**: conceptualization; data curation; investigation; methodology; software. **Niloufar Ahmadi**: data curation; formal analysis; investigation; writing – original draft; writing – review & editing. **Reza Mohseni Ahangar**: conceptualization; methodology; writing – review & editing. **Ali Asadolahzadeh**: conceptualization; data curation. **Mahmoud Sadeghi Haddad‐Zavareh**: conceptualization; methodology; validation; writing – review & editing. **Fatemeh khoshkhou**: data curation. **Zeynab Qolami**: Data curation. **Neda Mahdinezhad Gorji**: Data curation. **Mouloud Agajani Delavar**: conceptualization; formal analysis; methodology; project administration; validation; writing – review & editing.

## CONFLICT OF INTEREST STATEMENT

The authors declare no conflicts of interest.

## ETHICS STATEMENT

The study was approved by Iran University of Medical Sciences [IR.MUBABOL.HRI.REC.1400.078]. Consent for participant obtained from all patients in hospital admission. Consent for publication obtained from that patients or family of patients.

## Data Availability

All data generated or analyzed during this study are included from preliminary studies are available from the corresponding author on reasonable request.
